# EMMPRIN expression is associated with metastatic progression in osteosarcoma

**DOI:** 10.1186/s12885-021-08774-9

**Published:** 2021-09-26

**Authors:** Han-Soo Kim, Ha Jeong Kim, Mi Ra Lee, Ilkyu Han

**Affiliations:** 1 Department of Orthopaedic Surgery, Seoul National University Hospital, 101 Daehak-ro Jongno-gu, Seoul, 03080 South Korea; 2 Department of Orthopaedic Surgery, Seoul National University College of Medicine, Seoul, South Korea

**Keywords:** EMMPRIN, Osteosarcoma, Matrix metalloproteinase, Invasion, Metastasis

## Abstract

**Background:**

Extracellular matrix metalloproteinase inducer (EMMPRIN), a cell-surface glycoprotein, is overexpressed in several cancer types. EMMPRIN induces a metastatic phenotype by triggering the production of matrix metalloproteinase proteins (MMPs) such as MMP1 and MMP2, and vascular endothelial growth factor (VEGF) in cancer cells and the surrounding stromal cells. The purpose of this study was to investigate the expression and role of EMMPRIN in osteosarcoma.

**Methods:**

The level of EMMPRIN expression was evaluated using reverse transcriptase polymerase chain reaction (RT-PCR) in 6 tumor-derived osteosarcoma cell lines and compared with that in normal osteoblasts. To study the prognostic significance of EMMPRIN expression, immunohistochemistry was carried out in prechemotherapy biopsies of 54 patients. siRNA knockdown of EMMPRIN in SaOS-2 cells was conducted to explore the role of EMMPRIN. To study the role of EMMPRIN in tumor-stromal interaction in MMP production and invasion, co-culture of SaOS-2 cells with osteoblasts and fibroblasts was performed. Osteosarcoma 143B cells were injected into the tail vein of BALB/c mice and lung metastasis was analyzed.

**Results:**

EMMRIN mRNA expression was significantly higher in 5 of 6 (83%) tumor-derived cells than in MG63 cells. 90% of specimens (50/54) stained positive for EMMPRIN by immunohistochemistry, and higher expression of EMMPRIN was associated with shorter metastasis-free survival (*p* = 0.023). Co-culture of SaOS-2 with osteoblasts resulted in increased production of pro-MMP2 and VEGF expression, which was inhibited by EMMPRIN-targeting siRNA. siRNA knockdown of EMMPRIN resulted in decreased invasion. EMMPRIN shRNA-transfected 143B cells showed decreased lung metastasis in vivo.

**Conclusions:**

Our data suggest that EMMPRIN acts as a mediator of osteosarcoma metastasis by regulating MMP and VEGF production in cancer cells as well as stromal cells. EMMPRIN could serve as a therapeutic target in osteosarcoma.

## Background

Osteosarcoma is one of the most frequent malignant tumors, which often appears in young persons and expresses high cancerous and metastatic potential [1, 2]. Furthermore, it has been known to have a multiplied trend to metastasize [1, 3, 4]. The outcome for osteosarcoma patients with metastatic disease has not improved, thus, new therapies are essential [5]. The modulation of tumor suppressor genes and oncogenes is significant in the osteosarcoma progression [6–8].

Despite the enhancement of survival in patients with localized osteosarcoma, those with metastatic disease still carry a poor prognosis. As a result, identification of factors contributing to metastasis is needed. Biological characteristics of a malignant tumor are invasion and metastasis. The important step in the invasion and metastasis is the degradation of the extracellular matrix (ECM), and the matrix metalloproteinase proteins (MMPs) are the key players in the degradation of the ECM. Furthermore, the overexpression of MMP in malignancies is a contributing factor in metastasis, invasiveness, migration, and angiogenesis [9].

Extracellular matrix metalloproteinase inducer (EMMPRIN), known as basigin (BSO) and CD147, is a transmembrane glycoprotein of the immunoglobulin superfamily and plays manifold roles in physiological as well as in pathological conditions. It is strongly expressed in several types of cancers and activates adjacent stromal or tumor cells to induce matrix metalloproteinase (MMP). Additionally, EMMPRIN stimulates the vascular endothelial growth factor (VEGF) which is directly involved in angiogenesis. These findings suggest that EMMPRIN is an exciting therapeutic target in many cancer types. However, the role of EMMPRIN in the pathogenesis of osteosarcoma is unclear.

In this study, we assessed the levels of EMMPRIN gene expression and MMP activation in osteosarcoma cells from human samples and examined the role of EMMPRIN in the MMPs expression and invasiveness by EMMPRIN siRNA transfection. Our results suggest that EMMPRIN regulates MMP activation via tumor-stromal interaction and promotes invasiveness and metastasis in osteosarcoma. Furthermore, a current study confirmed that EMMPRIN silencing could inhibit osteosarcoma cell growth and invasion both in vitro and in vivo.

## Methods

### Cell lines

Osteosarcoma cells were isolated from four sources to be used in the investigations: [1] pre-chemotherapy tumor samples of 6 patients who underwent treatment in the authors’ hospital from March 2003 to April 2004; [2] an established osteosarcoma cell line of Saos-2 (American Type Culture Collection (ATCC) Nu HTB-85) [9]; [3] hFOB, from immortalized fetal osteoblasts (ATCC Nu CRL-11372) [10], and [4] MG-63 (ATCC Nu CRL-1427) [11]. All cell lines were purchased from the American Type Culture Collection, and cultured according to their guidelines [9–11]. Primary osteosarcoma cell lines were prepared as previously described in Kang et al.’s paper [12]. In brief, samples were minced and incubated at 37 °C in DMEM with 0.2% proteinase (Sigma-Aldrich, St. Louis, MO) and 0.2% collagenase (Sigma-Aldrich, St. Louis, MO). The supernatant was filtered and centrifuged at 1500 rpm for 5 min. The cell pellet was re-suspended in DMEM with 10% fetal bovine serum (FBS) and seeded in 100 mm plates at 2 × 10^6^ cells. After 3–5 days, the cells were sub-cultured. Second - or third-passage cells were selected for the experiments.

### Transfection and co-culture of SaOS-2 and hFOB

SaOS-2 cells were cultured in MEM with Earle’s salts or McCoy’s 5A medium with 10% FBS. For the experiments, cells were plated at density of 2.0 × 10^5^ cells/cm^2^ with medium changes done twice a week at 37 °C in a humidified atmosphere with 5% CO_2_. SaOS-2 cells were transfected with Silencer Negative Control siRNA (Life Technologies, Carlsbad, CA, USA) as a control and EMMPRIN siRNA (5′-UUC UGA CGA CUU CAC AGC CUU CAC U-3′, Invitrogen, USA) using Lipofectamine-2000 (Invitrogen, San Diego, CA, USA). Cells were then incubated for 24 h. hFOB cells were cultured in Dulbecco’s modified Eagle’s medium/F12 (DMEM/F12) supplemented with 10% FBS and G418 (0.3 mg mL^− 1^, Gibco, Carlsbad, CA, USA) and maintained in a humidified atmosphere with 5% CO_2_ at 34 °C. The cells were digested with 0.25% trypsin and split at a 1:3 ratio. hFOB was used in the differentiation phase described by ATCC CRL 11372. Confluent hFOB cells were seeded above TransWell® 12 well inserts (Corning Costar Corporation, Cambridge, MA, USA) with a pore size of 0.4 μm for incubation in conditioned medium. When hFOB monolayers were established, they were washed with DMEM. Supernatants from upper and the lower TransWell® 12 well chambers were separately collected at 24 h.

### Reverse transcriptase polymerase chain reaction (RT-PCR)

To examine the expression of EMMPRIN mRNA in osteosarcoma, RT-PCR was performed in 6 osteosarcoma primary cell lines derived from pre-chemotherapy tumors of patients. Total RNA was extracted by TRIzol reagent (Invitrogen, Carlsbad, CA). RNA quantity and quality were assessed using the Nanodrop-ND-1000 (Nanodrop Technologies, Wilmington, DE, USA). cDNA was synthesized with the Reverse Transcription System (Promega, Madison, Wisconsin, USA). The primers for PCR amplification were: CD147, sense: 5′-GCAGCGTTGGAGGTTGT-3′, antisense: 5′-AGCCACGGATGCCCAGGAAGG-3′; GAPDH (internal control), forward primer 50-CATGAGAAGTATGACAACAGCCT-30, reverse primer 50-AGTCCTT CCACGATACCAAAGT-30. The cycling program was pre-set at 94 °C for 5 min for degeneration, and 30 cycles for 30 s at 94 °C, 55 °C for 60 s, and 72 °C for 30 s, and finishing with for 10 min at 72 °C for elongation. The RT–PCR products were electrophoresed in 2% agarose gel with ethidium bromide [13].

### Gelatin Zymography

The tumor microenvironment is critical for the proliferation and invasion of the tumor. Matrix metalloproteinases (MMPs) is crucial in the tumor microenvironment [14]. SaOS-2 cells and hFOB cells were plated in 6-well plates to either culture alone or in a co-culture system. For zymography analysis to detect MMP2 expression, cells were cultured in serum-free media for 48 h [15, 16]. Conditioned media were centrifuged and stored at − 70 °C. Protein lysates were prepared in 50 mM Tris-HCl containing 150 mM NaCl and 0.1% NP-40 with 2 g/ml pepstatin, leupeptin (Roche Diagnostics, Mannheim, Germany) and aprotinin (Sigma Chemical Co., St. Louis, MO). The gelatin zymograms were calibrated with gelatinase standard by capillary whole blood (Chemicon, Hampshire, United Kingdom). Staining of gels were done with 0.2% Coomassie brilliant blue R-250 (Bio-Rad Laboratories, Hercules, CA, USA). Reconstituted lyophilized human pro-MMP2 was used as a positive control (Biotrak MMP2 activity assay kit, GE Healthcare). Gelatinase activity was observed as clear regions in blue gels. The bands were quantified considering both the intensity and the extension area, using an image analyzer system with ImageJ software version 1.49 (National Institutes of Health, Bethesda, Maryland, USA).

### Elisa

EMMPRIN is known to stimulate tumor angiogenesis via VEGF [17, 18]. To study the possible role of EMMPRIN in angiogenesis, we co-cultured osteoblasts and SaOS-2 cells. Quantitation of VEGF in co-culture supernatants was done using ELISA. Cells infected with mock siRNA were cultured in 6-well plates (1.0 × 10^5^ cells per well). After 48 h, the medium was replaced with 1 mL of serum-free DMEM. After collecting the conditioned medium 3 days later, and the concentration of secreted VEGF protein was examined by ELISA kit (R&D Systems, Minneapolis, MN, USA) [13].

### Western blotting analyses

Transfected cells were incubated using fibronectin plate for 4 h, and then lysed with buffer (NP-40 1%, 150 mM NaCl, 50 mM Tris pH 7.5, and proteinase inhibitor cocktail). Lysates were resolved by SDS-PAGE and then transferred onto nitrocellulose membrane. The membranes were probed with primary antibodies against EMMPRIN (Cell Signaling, #13287S, Lot1) after incubating in a blocking buffer. The labeling was observed using peroxidase-conjugated secondary antibodies and an ECL kit (Pierce, Thermo Fisher Scientific, Waltham, MA, USA) [19].

### Matrigel invasion assay

Invasion assays were performed using a BD BioCoat Matrigel Invasion Chamber (BD, New Jersey, USA) (pore size, 8 μm). EMMPRIN-siRNA-transfected SaOS-2 cells were introduced into the upper compartment in transwell chambers, and the lower compartment contained hFOB cells. Cells were incubated for 24 h, fixed, stained with 0.5% crystal violet and then counted under light microscope.

### In vivo metastasis assay

To test if EMMPRIN plays an important role in osteosarcoma metastasis in vivo, the osteosarcoma cell line 143B was injected in the tail vein of BALB/c mice. The mice were sacrificed at 8 weeks post-injection. Four-week-old male BALB/c nude mice were obtained from Central Lab. Animal Inc. (Seoul, Korea) and maintained under standard conditions until the experiments were performed. The animals were maintained at the animal facility of the Seoul National University Hospital under guidelines prior to the grouping and experiments. A total of 15 BALB/c nude mice were randomized into 3 groups: [1] normal, [2] 143 cells transfected with an ad mock shRNA vector (Control), and [3] 143 cells transfected with the ad EMMPRIN shRNA vector. Experiments were approved by the Institutional Animal Care and Use Committee of Seoul National University Hospital (approval number 10–0075). One anti-EMMPRIN sequence (5-GTCGTCAGAACACATCAAC-3) or a scrambled sequence was inserted into the plasmid vector pAdEasy-1 (Addgene). They were designated as pAdEasy-1-shRNA and pAdEasy-1 scramble shRNA, respectively. Osteosarcoma cell line 143B was transfected with EMMPRIN shRNA. EMMPRIN shRNA transfected 143B cells were harvested with trypsin, and then resuspended in serum-free RPMI, and injected in the tail vein (1 × 10^5^/0.2 mL) of 5 nude mice per group. Health of the animals was monitored daily, and body weights were measured weekly throughout the study period. Anesthesia was performed with isoflurane inhalation as well as ketamine (10 mg/kg) and medetomidine (0.1 mg/kg) injection. All surgical procedures were performed under sterile conditions. Secondary euthanasia method for cervical dislocation was also performed. The mice were sacrificed by CO_2_ inhalation at 8 weeks post-injection. Harvested tissues were preserved in Bouin’s fixative, embedded in paraffin, sectioned (4 μm), and stained with hematoxylin and eosin (H&E). Examination of the histological sections was performed using Nikon Eclipse Ci microscope (Nikon Corp., Tokyo, Japan) by a digital camera (Nikon digital sight, DS-2Mv) and the automatic exposure and iSolution Lite software for microscopic images. The tumor lengths and widths were measured by a perpendicular tumor diameter, with the tumor volume being calculated using the following formula: width^2^ × length/2 [20].

### Immunohistochemistry and clinical outcome

To examine EMMPRIN expression in a larger cohort of patients, immunohistochemistry was performed in pre-chemotherapy osteosarcoma specimens from 52 patients. Pre-chemotherapy biopsy specimens were used to analyze the EMMPRIN expression by immunohistochemistry [21]. Osteosarcoma specimens derived from patients who underwent operation at Seoul National University Hospital with clinical annotation of more than 1 year follow-up were used. Briefly, 4 μm sections in paraffin were de-paraffinized using xylene and rehydrated in alcohol solution. Antigen retrieval was performed by pretreating the slides in citrate buffer. To suppress binding of nonspecific antigen-antibody, tissues were treated with a blocking solution of 10% nonimmune serum and then incubated overnight with mouse monoclonal antibody of EMMPRIN (Abcam, #ab188190, GR3250057–5) and stained with the avidin-biotin-peroxidase complex method (Vectastain ABC kit, Vector Laboratories, Burlingame, CA). Staining reactions were interpreted with parallel-processed control slides consisting of esophageal cancer known to express EMMPRIN as the positive control, and with the negative control after replacing primary antibody with Tris-buffered saline [22]. The degree of EMMPRIN expression was evaluated by one of the investigators blinded to the patients’ clinical data. The expression of EMMPRIN was semi-quantitatively assessed by the extent (not intensity) of staining. After finding the areas showing the most tumor cells at × 40 magnificatio, cells were counted under × 100 magnification. Samples were graded into high and low expression groups by the percentage of positively staining cells among tumor cells with a cutoff value of 50% [23]. Examination of the histological sections was performed using Nikon Eclipse Ci microscope (Nikon Corp., Tokyo, Japan) and the automatic exposure and iSolution Lite software for microscopic images.

All patients had localized high-grade osteosarcoma of the extremities at the time of diagnosis. Standard treatment was given, consisting of neoadjuvant chemotherapy and primary resection followed by adjuvant chemotherapy. The response to chemotherapy was assessed according to the Huvos criteria [17]. Overall survival was defined as the time from osteosarcoma diagnosis to the date of either death or the last hospital visit. We defined metastasis-free survival as the time from osteosarcoma diagnosis to the development of metastasis. The minimum follow-up was 12 months (range, 12–110 months; mean, 42 months). The mean follow-up of the survivors was 50 months. During the follow-up period, local recurrence was observed in 11 patients, and metastasis in 20. Fourteen of the 52 patients died of disease, 37 patients showed no evidence of disease, and 5 remained alive with disease at the last follow-up.

### Statistical analysis

Statistical analyses were performed using the SPSS 12.0 software. Survival curves were calculated by the Kaplan–Meier method, with the differences in survival calculated using the log-rank test. Correlations between EMMPRIN expression and clinical factors were evaluated using Pearson’s x^2^ test (*P* < 0.05). Uni- and multi-variate analyses were performed using the Cox proportional hazards model.

## Results

### EMMPRIN mRNA expression in osteosarcoma cells

**Fig. 1:**
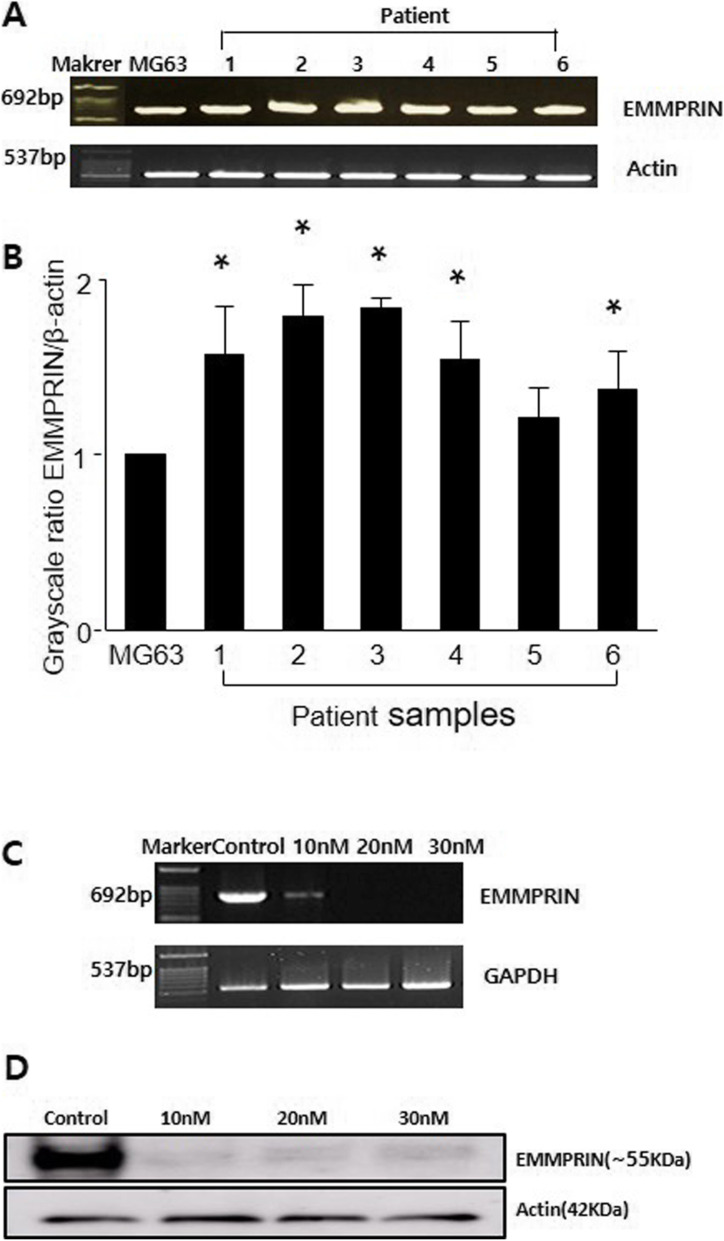
EMMPRIN expression in osteosarcoma by reverse transcription-PCR and Western blot. **a** Reverse transcription-PCR products of total RNA in tumor-derived osteosarcoma cells from 6 patients. **b** Comparison of grayscale ratio of EMMPRIN/β-actin in tumor-derived osteosarcoma cells and MG63. *, *p* < 0.05 compared with MG63. **c** EMMPRIN mRNA expression after transfection of SaOS-2 cell by EMMPRIN-targeting siRNA. Data shown are representative images of individual cell lines from three separate experiments. **d** Western blot analysis of EMMPRIN protein expressions. β-actin was used as loading control

The EMMRIN mRNA expression level was significantly higher in 5 of 6 (83%) tumor-derived cells compared to MG63 (Fig. 1A, B). EMMPRIN mRNA and protein levels were significantly downregulated by siRNA transfection in comparison to control siRNA transfected cells (Fig. 1C, D). siRNA (30 nM) was used for further experiments in terms of its lowest expression.

### EMMPRIN knockdown and MMP2 activity

**Fig. 2:**
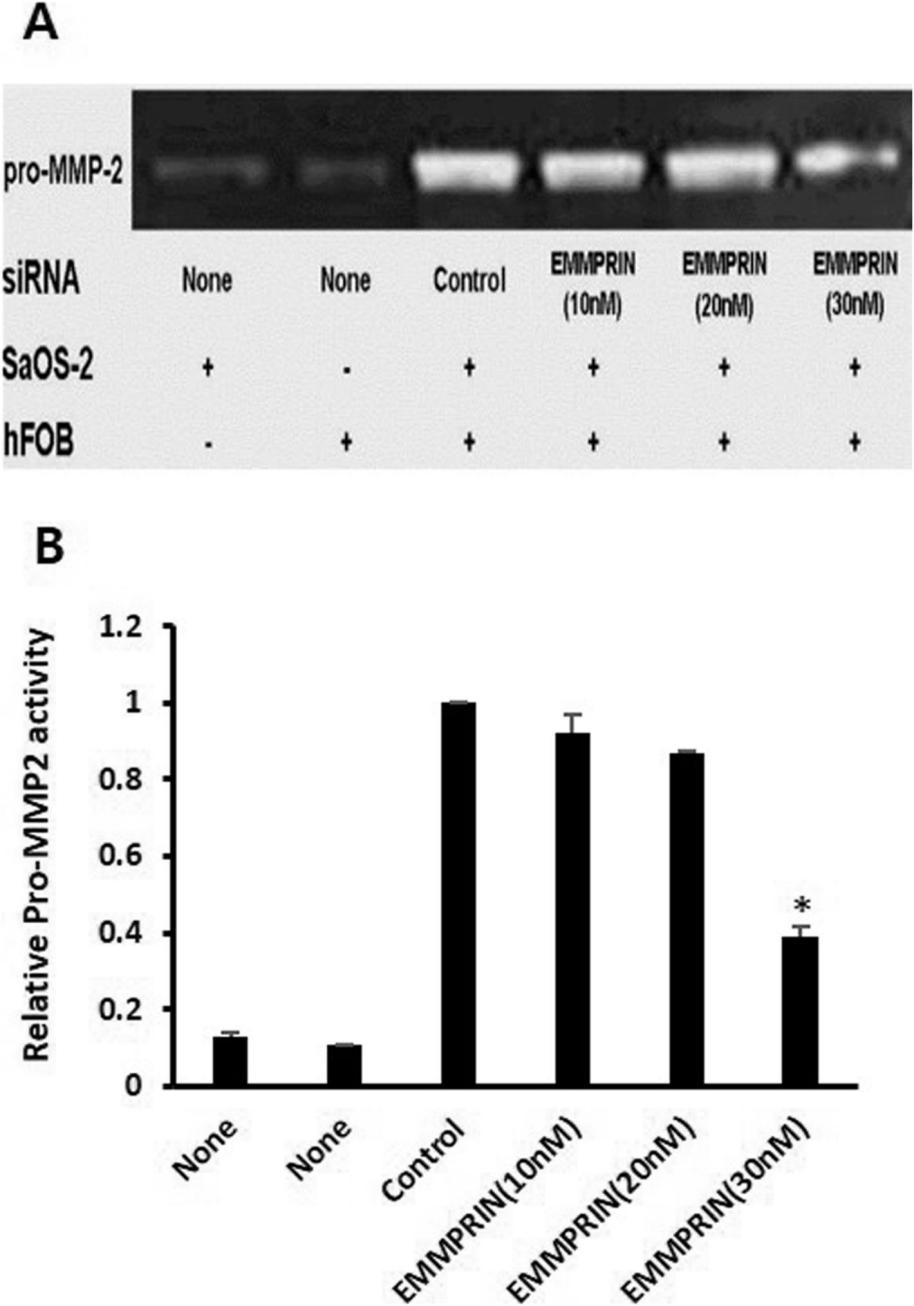
Reversal of MMP2 stimulation in co-cultures of SaOS-2 and osteoblast by EMMPRIN siRNA transfection. **a** SaOS-2 and osteoblast showed weak gelatinolytic band for pro-MMP2 when cultured alone (Lanes 1, 2). When osteoblast and SaOS-2 were co-cultured, enhanced stimulation of pro-MMP2 was observed (Lane 3). This stimulation was reversed by transfection of SaOS-2 with EMMPRIN-targeting siRNA (Lanes 4–6). Data shown are representative images of individual cell lines from three separate experiments. **b** Comparison of grayscale ratio of gelatinolytic band for pro-MMP2 *, *p* < 0.05 compared with control

[23] To study the role of EMMPRIN in tumor-stromal interaction, we co-cultured SaOS-2 cells with osteoblasts (hFOB). Co-culturing of osteoblasts and SaOS-2 enhanced the stimulation of pro-MMP2. This stimulation was reversed by transfection of SaOS-2 cells with EMMPRIN-targeting siRNA (Fig. 2A). The grayscale ratio of MMP2 activity in co-cultures of 30 nM EMMPRIN siRNA transfected SaOS-2 cells with hFOB cells was significantly lower than that of the control (*p* = 0.01) (Fig. 2B). These results show that EMMPRIN inhibition results in decreased MMP2 activity in co-cultures of SaOS-2 cells with hFOB cells.

### EMMPRIN knockdown and VEGF production

**Fig. 3:**
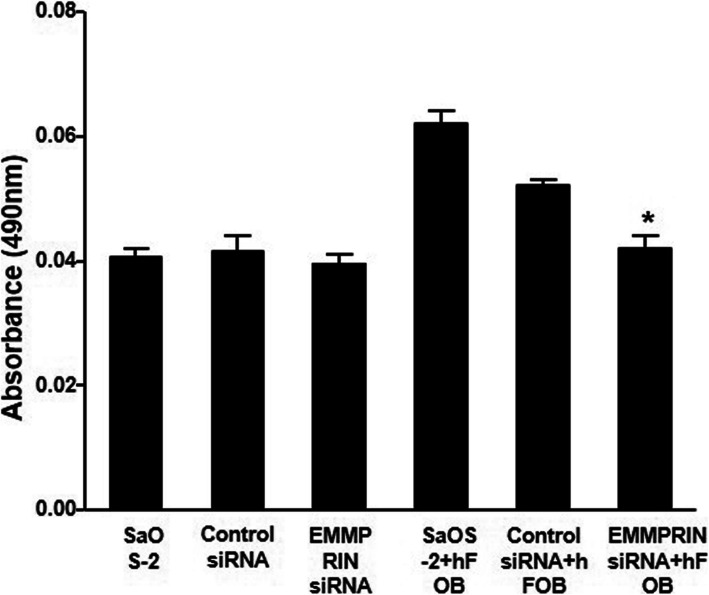
Decrease of VEGF production in co-cultures of SaOS-2 and osteoblast by EMMPRIN siRNA transfection. When osteoblasts and SaOS-2 cells were co-cultured, VEGF expression was increased compared to the SaOS-2-only culture. Transfection of SaOS-2 cells with EMMPRIN-targeting siRNA resulted in a decrease in VEGF expression. * *p* < 0.05 compared with cells transfected with control siRNA

[17, 18] VEGF expression was increased in the co-culture compared to the SaOS-2-only culture. Transfection of SaOS-2 with EMMPRIN-targeting siRNA resulted in a decrease in VEGF expression (Fig. 3).

### EMMPRIN knockdown and invasion

**Fig. 4:**
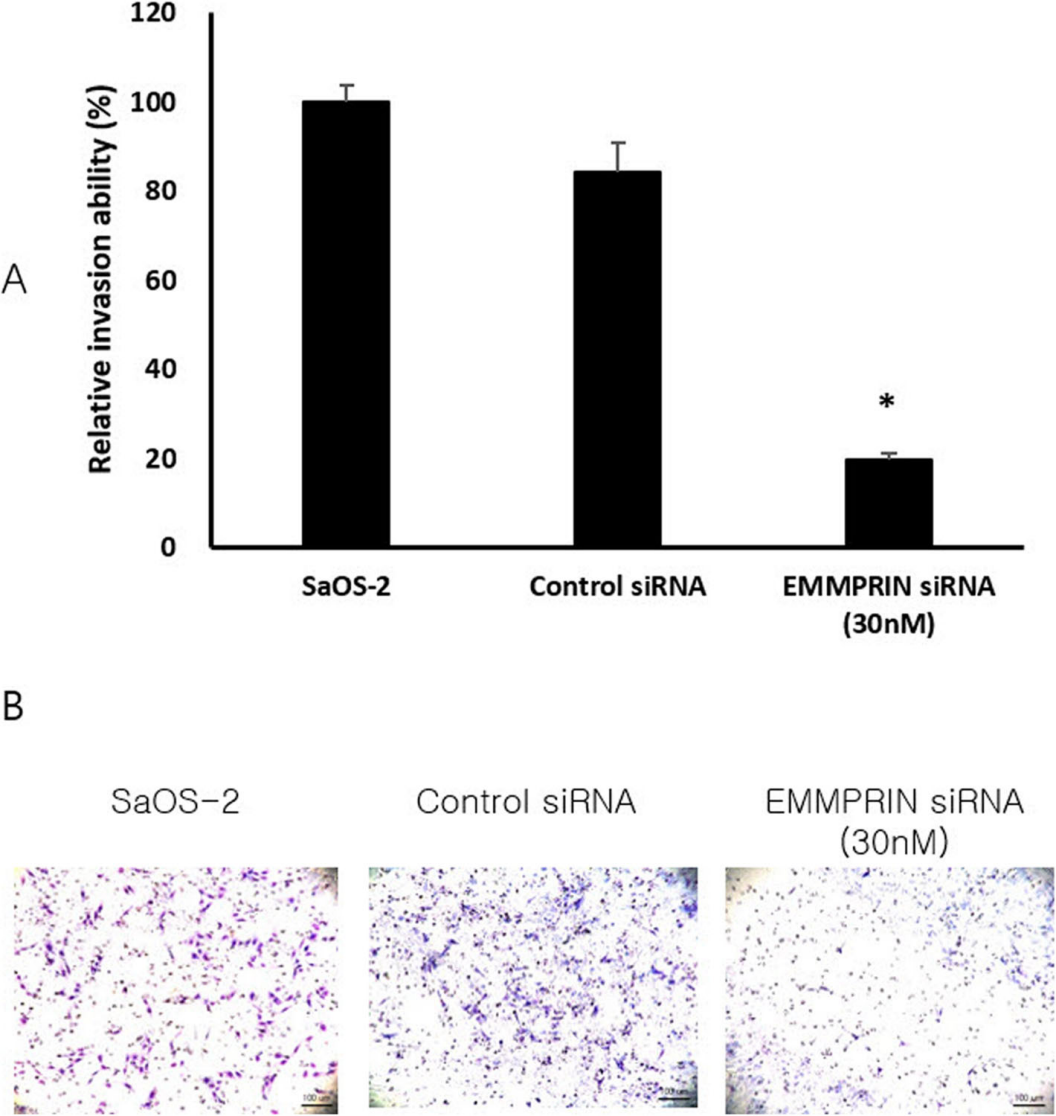
Decrease in invasion activities of SaOS-2 cells transfected by EMMPRIN siRNA. Matrigel invasion assays after transfection of the EMMRIN siRNA into SaOS-2 cells for 24 h. Following incubation, the membranes were removed from the inserts and mounted on slides. a. The number of invading cells was counted under a light microscope. The Matrigel assay was performed in triplicates. * *p* < 0.05 compared with cells transfected with control siRNA. b. Representative images of Matrigel invasion assay. Scale bar, 100 μm

The invasion of SaOS-2 cells treated with 0 and 30 nM EMMPRIN siRNA was detected by the transwell chamber assay after treatment for 24 h. The number of cells crossing the chamber were statistically lower in cells transfected with 30 nM EMMPRIN-siRNA than in cells transfected with control-siRNA (25.2 ± 3.5 cells vs. 5.0 ± 1.4 cells *P* = 0.048) (Fig. 4).

### EMMPRIN silencing and metastatic potential in vivo

**Fig. 5:**
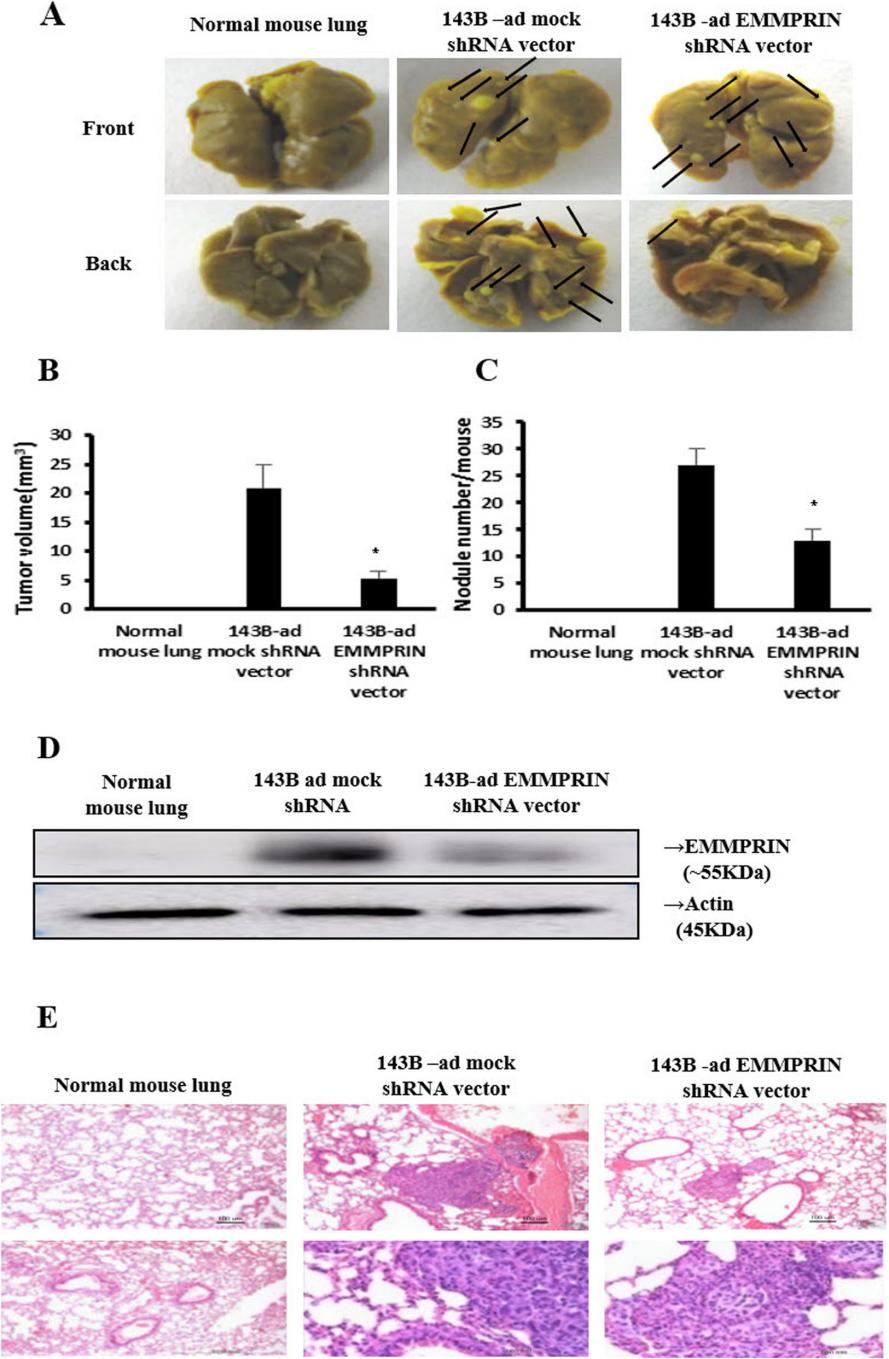
EMMPRIN silencing inhibits the metastatic nodes in the lungs. **a** Metastasis assay by tail vein injection of shRNA transfected 143B cells in BALB/c mice. Lungs were excised for examination at 8 weeks post-injection. The number of lung metastatic nodes was assessed by Bouin’s stain. Arrows indicate visible lung surface metastases. **b** Tumor volume and **c** Number of nodules at the study end point. **d** 143B cells were transfected with mock shRNA and EMMPRIN shRNA for 48 h, and cell lysates were analyzed by western blot using anti-EMMPRIN antibody. β-actin was used as a loading control. **e** Microscope images by H&E staining show that EMMPRIN silencing significantly inhibits the lung metastatic nodes after inoculation with 143B, 2 × 10^5^ cells/ 200 ul. *, *p* < 0.05 compared with cells transfected with control siRNA. Scale bar, 100 μm. Original magnification, × 10 and × 40

The tumor volume was significantly reduced in EMMPRIN shRNA transfected 143B cells as compared to the corresponding mock shRNA transfected 143B cells in vivo (5.3 ± 1.2 mm^3^ vs. 20.9 ± 3.9 mm^3^, *p* = 0.002) (Fig. 5A,B) The number of nodules was also reduced in EMMPRIN shRNA transfected cells (13.2 ± 2.00 vs. 27.0 ± 3.0, *p* = 0.001) (Fig. 5A,C). These results show that EMMPRIN regulates lung metastasis in osteosarcoma. We used shRNAs to show that EMMPRIN expression was significantly decreased in 143B cells by western blotting (Fig. 5D), whereas its mock shRNA enhanced protein production in 143B cells. H&E staining showed typical tumor morphologies, that is, the nuclei were large, deeply stained, and the cells were closely arranged in vivo (Fig. 5E).

### EMMPRIN expression and clinical outcome

**Fig. 6:**
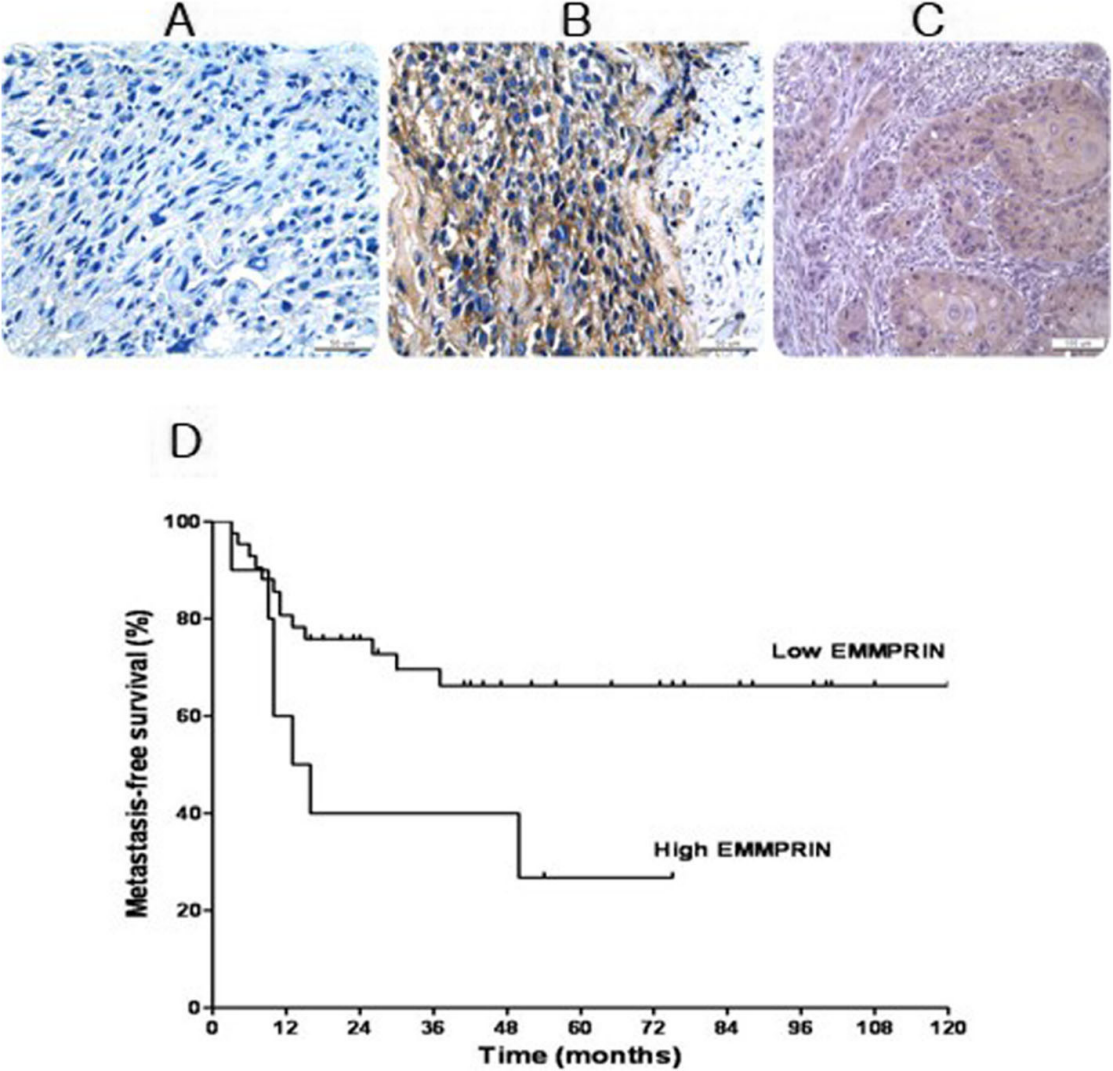
EMMPRIN expression in osteosarcoma by immunohistochemical staining and metastasis-free survival. Representative case for **a** low EMMPRIN expression. **b** high EMMPRIN expression and **c** esophageal cancer, a positive control. Scale bar, 50 μm. **d** Metastasis-free survival of 52 patients with osteosarcoma in relation to EMMPRIN expression. The survival time in individual patients was determined and the survival curves of patients with different expression of EMMPRIN were generated by Kaplan-Meier method

Osteosarcoma cells showed strong positivity for EMMPRIN with accentuation along the cell membrane. EMMPRIN was expressed in 96% of cells (*n* = 50) as indicated by immunohistochemistry (Fig. 6A, B). Of the 52 cases, 10 (19%) showed the EMMPRIN expression of more than 50% of the tumor cells (high expression) and 42 cases (81%) less than 50% (low expression). Of the 10 cases with high expression, 7 patients developed metastases, whereas only 13 of 42 cases with low expression developed metastases. Patients with high EMMPRIN expression had significantly worse metastasis-free survival (*p* = 0.023) (Fig. 6D).

## Discussion

In our study, we verified the protein expression mechanism of EMMPRIN, MMP2, and VEGF in osteosarcoma by immunohistochemical staining and molecular experiments, and characterized the expression patterns of EMMPRIN with the help of clinicopathological discoveries and recurrence-free survival in 52 patients with osteosarcoma. Moreover, the expression of EMMPRIN, as shown by immunohistochemistry, was examined in 90% of osteosarcoma tissues. Patients with high EMMPRIN expression had significantly worse metastasis-free survival. This finding was consistent with the results for other human tumors [18, 24, 25]. In line with our study, Zhou et al. reported that expression of EMMPRIN was detected in 70% of patients with osteosarcoma. EMMRPIN levels were elevated in ostesarcoma patients with advanced disease and worse response to chemotherapy as compared to those with less advanced stage and better response to chemotherapy [23]. Variable expression of EMMPRIN was found in 65% of human prostate cancer tissues and correlated significantly with progression parameters [24]. Increased expression of EMMPRIN was monitored in primary oral squamous cell carcinoma and metastatic oral squamous cell carcinoma specimens. Moreover, a strong EMMPRIN expression was found in more than 90% of the cells in the carcinomas-in-situ and early invasive squamous cell carcinoma [25]. Several studies have reported high levels of EMMPRIN expression is involved in many cancers [13, 26–36]. EMMPRIN expression has been also considered as a marker of a poor prognosis for breast cancer patients [34]. Ueda et al. demonstrated that high EMMPRIN expression was associated with more recurrence in endometrial cancer [35].

It is widely noted that angiogenesis in the EMMPRIN-MMP-VEGF system has a significant role in tumor progression by cancer stroma interaction [37]. Recent studies have shown that VEGF and MMP production are stimulated immediately by elevated EMMPRIN expression in tumor cells as well as stromal cells in breast cancer [38]. Moreover, to regulate VEGF production in an MMP-dependent manner, in vivo soluble VEGF is increased or biologically active angiogenic growth factor is released by tumor-derived MMP [18]. Cell adhesion, cytoskeleton reorganization, and cell invasion in prostate cancer cells are controlled by EMMPRIN [36]. In bladder, prostate, and gastric cancer, a significant inhibitory effect is exhibited when transfected with EMMPRIN siRNA [13, 39, 40]. In this study, the suppression of cell proliferation, invasion, and metastasis in osteosarcoma specimens was demonstrated after the transfection of the EMMPRIN siRNA. We observed that EMMPRIN knockdown inhibited the secretion of MMP2, the expression of VEGF, and invasion and metastasis of osteosarcoma. The results are consistent with other reports suggesting the inactivation of MMP2 and VEGF by EMMPRIN knockdown inhibits cancer migration and invasion [40–42]. Indeed, in our study, EMMPRIN-targeting siRNA inhibited proliferation and invasion of osteosarcoma cells.

To examine the effect of EMMPRIN silencing on osteosarcoma cell metastasis, we injected EMMPRIN shRNA transfected 143B cells in nude mice and assessed the presence of metastatic nodules in the lung. The EMMPRIN shRNA vector suppressed the expression of EMMPRIN in pancreatic cancer cells, reducing the invasion and metastasis in vitro and in vivo [43]. The expression of EMMPRIN mRNA and protein in MCF-7 cells was reduced with the EMMPRIN-shRNA lentivirus, which has been verified through EMMPRIN shRNA in breast cancer [44]. The expression levels of EMMPRIN mRNA and protein in MCF-7 cells were significantly decreased after infection with the EMMPRIN-shRNA lentivirus, which was proven through EMMPRIN shRNA in breast cancer MCF-7 cells using the lentivirus-induced RNAi technique to investigate the changes in breast cancer proliferation potential under conditions of EMMPRIN gene deletion. Li et al. used an shRNA vector to lower EMMPRIN expression in the colorectal cancer cell line HT29 and found that levels of EMMPRIN mRNA and protein were reduced in vitro and in vivo [45]. Lung metastases are associated with poor prognosis in patients with osteosarcoma. The underlying molecular mechanisms of lung metastasis remain to be elucidated. In the present study, we found that knockdown of EMMPRIN inhibited lung metastasis.

## Conclusions

In conclusion, EMMPRIN overexpression may play an important role in the metastasis of osteosarcoma and could be a potential therapeutic target of osteosarcoma.

## Data Availability

The datasets used and/or analysed during the current study are available from the corresponding author on reasonable request.

## Abbreviations

OS: Osteosarcoma
ECM: Extracellular matrix
MMPs: Matrix metalloproteinase proteins
TIMPs: Tissue inhibitors of metalloproteinases
EMMPRIN: Extracellular matrix metalloproteinase inducer
VEGF: Vascular endothelial growth factor
RT-PCR: Reverse transcriptase polymerase chain reaction
